# Comparative Genomics Reveals a Single Nucleotide Deletion in *pksP* That Results in White-Spore Phenotype in Natural Variants of *Aspergillus fumigatus*

**DOI:** 10.3389/ffunb.2022.897954

**Published:** 2022-06-07

**Authors:** John G. Gibbons, Paolo D’Avino, Shu Zhao, Grace W. Cox, David C. Rinker, Jarrod R. Fortwendel, Jean-Paul Latge

**Affiliations:** ^1^Department of Food Science, University of Massachusetts, Amherst, MA, United States; ^2^Molecular and Cellular Biology Graduate Program, University of Massachusetts, Amherst, MA, United States; ^3^Organismic & Evolutionary Biology Graduate Program, University of Massachusetts, Amherst, MA, United States; ^4^Department of Biological Sciences, Vanderbilt University, Nashville, TN, United States; ^5^Department of Clinical Pharmacy and Translational Science, University of Tennessee Health Science Center, Memphis, TN, United States; ^6^Aspergillus Unit, Institut Pasteur, Paris, France

**Keywords:** Aspergillus, albinism, genomics, pksP gene, 1,8-Dihydroxynaphthalene (DHN) melanin

## Abstract

*Aspergillus fumigatus* is a potentially deadly opportunistic human pathogen. *A. fumigatus* has evolved a variety of mechanisms to evade detection by the immune system. For example, the conidium surface is covered in a layer of 1,8-dihydroxynaphthalene (DHN) melanin which masks the antigen macrophages use for recognition. DHN melanin also protects conidia from ultraviolet radiation and gives *A. fumigatus* conidia their characteristic green-grayish color. Here, we conducted genomic analysis of two closely related white-spore natural variants of *A. fumigatus* in comparison to two closely related green-spore isolates to identify a genetic basis of the white-spore phenotype. Illumina whole-genome resequencing data of the four isolates was used to identify variants that were shared in the white-spore isolates and different from both the green-spore isolates and the Af293 reference genome (which is also a green-spore isolate). We identified 4,279 single nucleotide variants and 1,785 insertion/deletions fitting this pattern. Among these, we identified 64 variants predicted to be high impact, loss-of-function mutations. One of these variants is a single nucleotide deletion that results in a frameshift in *pksP* (*Afu2g17600*), the core biosynthetic gene in the DHN melanin encoding gene cluster. The frameshift mutation in the white-spore isolates leads to a truncated protein in which a phosphopantetheine attachment site (PP-binding domain) is interrupted and an additional PP-binding domain and a thioesterase domain are omitted. Growth rate analysis of white-spore and green-spore isolates at 37°C and 48°C revealed that white-spore isolates are thermosensitive. Growth rate of *A. fumigatus* Af293 and a *pksP* null mutant in the Af293 background suggests *pksP* is not directly involved in the thermosensitivity phenotype. Further, our study identified a mutation in a gene (*Afu4g04740)* associated with thermal sensitivity in yeasts which could also be responsible for the thermosensitivity of the white-spore mutants. Overall, we used comparative genomics to identify the mutation and protein alterations responsible for the white-spore phenotype of environmental isolates of *A. fumigatus*.

## Introduction

*A. fumigatus* is an opportunistic human pathogen responsible for the highest number of deaths and for the second highest number of infections of any fungal species (Latge, 1999; Brown et al., 2012). *A. fumigatus* primarily causes infections in individuals with compromised immune systems (Latge, 1999). Individuals with blood, bone marrow, and lymph node cancers, patients who have recently undergone solid organ transplantation surgery, and recipients of hematopoietic stem cell transplantation are at highest risk for invasive aspergillosis, the most serious and systemic form of *A. fumigatus* infection (Steinbach et al., 2012).

*A. fumigatus* has evolved a collection of immune evasion and immune adaptation strategies that occur at the conidial and hyphal developmental stages (van de Veerdonk et al., 2017). For instance, the conidium surface is covered in a hydrophobic protein layer and a layer of 1,8-dihydroxynaphthalene (DHN) melanin. These molecules mask the immunogenic carbohydrate β(1,3)-glucan, which immune cells use for recognition (Couger et al., 2018). In addition, DHN-melanin enables *A. fumigatus* conidia to resist phagocytosis (Latge et al., 2017). Indeed, the conidia of DHN-melanin mutants are phagocytosed at significantly higher rates than wild-type conidia (Luther et al., 2007).

DHN-melanin accounts for the greenish-gray color of *A. fumigatus* conidia and is synthesized by a six gene cluster on chromosome 2 (*abr2*, *abr1*, *ayg1*, *arp2*, *arp1* and *pksP*) (Heinekamp et al., 2012). PksP produces naphthopyrone from acetyl-CoA and malonyl-CoA. Naphthopyrone is next shortened by Ayg1, reduced by Arp2 and Arp1, and finally oxidatively polymerized by Abr2 to form DHN-melanin (Tsai et al., 1998; Tsai et al., 2001; Fujii et al., 2004; Sugareva et al., 2006; Heinekamp et al., 2012). Interestingly, single knockout mutants of *abr2*, *abr1*, *ayg1*, *arp2*, *arp1* and *pksP* produce slightly different spore color phenotypes, with *ΔpskP* resulting in a complete loss of pigmentation (white conidia) (Heinekamp et al., 2012).

White-spore variants of *A. fumigatus* are occasionally clinically or environmentally isolated (Sarfati et al., 2002; Balajee et al., 2004; Couger et al., 2018), and could lead to misidentification, as green conidia color is a conserved characteristic of *A. fumigatus* (Sugui et al., 2014). Here, we aimed to understand the genetic basis of two natural *A. fumigatus* isolates displaying the white-spore phenotype. We sequenced the genomes of these isolates and compared them to two closely related *A. fumigatus* isolates that produce the typical greenish-gray spores.

## Materials and Methods

### *A. fumigatus* Isolates, Culturing, DNA Extraction

*A. fumigatus* IP_23 (originally known as CBS 386.75) and IP_24 (originally known as CBS 110.46) were obtained from the Westerdijk Fungal Biodiversity Institute (KNAW) of the Netherlands (Sarfati et al., 2002). IFM47072 and IFM59985 are clinical isolates originally isolated from Japan (Zhao et al., 2021). Isolates were grown on potato dextrose agar (PDA) plates at 37°C for 72 hours. DNA was isolated directly from spores using the MasterPure yeast DNA purification kit following the manufacturer’s instructions, with several minor modifications, as previously described (Zhao et al., 2019), and as follows. Spores were collected with wash-store solution (49.9% glycerol, 50% potato dextrose broth and 0.1% Tween-20), centrifuged at 14,000 RPM for 5 minutes, and the supernatant was discarded. Next, 300 ml of yeast cell lysis solution was added to the spores along with 0.4 ml of 1.0 μm diameter silica beads. Cell lysis was carried out on a Biospec Mini-BeadBeater-8 at medium intensity for 8 minutes. One μl of RNase was added to the cell lysis solution and incubated at 65°C for 30 minutes. DNA purification was conducted according to the manufacturer’s instructions for the remainder of the protocol.

### Whole-Genome Sequencing and Assembly

IFM47072 and IFM59985 were previously sequenced and whole-genome Illumina data is available through the NCBI Sequence Read Archive under run accession numbers SRR11977809 and SRR11977822, respectively (Zhao et al., 2021). 150-bp paired-end libraries were constructed and sequenced for IP_23 and IP_24 by Novogene on an Illumina NovaSeq 6000 sequencer. Trim_Galore v0.4.2 was used to remove residual adaptor sequences and trim reads at low-quality sites using the parameters “–paired”, “–stringency 1”, “–quality 30” and “–length 50” (Martin, 2011). Read sets were then error corrected and assembled using SPAdes v3.13.1 with the “–careful” parameter and K-mer sizes of 55, 77, and 99 (Bankevich et al., 2012). Genome assemblies for IP_23 and IP_24 are available through NCBI accession numbers JALLAH000000000 and JALLAI000000000, respectively. Assembly quality, using the genome assembly as input, was assessed with BUSCO v5 using the “eurotiomycetes_odb10” ortholog set through the gVolante v2.0.0 server (Simao et al., 2015; Nishimura et al., 2017). Gene prediction was performed with Augustus v3.3.2 (Stanke and Waack, 2003) using the following parameters “–strand=both”, “–genemodel=complete”, “–gff3=on”, “–codingseq=1”, “–protein=1”, “–uniqueGeneId=true”, and “–species=aspergillus_fumigatus”.

### Phylogenetic Relationship of Isolates

We previously sequenced the genomes of IFM47072 and IFM59985 and inferred their evolutionary relationship with 74 additional isolates (Zhao et al., 2021). This analysis revealed four major *A. fumigatus* populations. We selected a minimum of four individuals from each population and included IFM47072, IFM59985, IP_23 (386.75) and IP_24 (110.46) to infer the phylogenetic relationship between samples. Briefly, GATK v4.0.6.0 was used to call SNVs across all samples relative to the Af293 reference genome using the best practice pipeline for “Germline short variant discovery” (Van der Auwera et al., 2013). Next, hard filtering was carried out to reduce false positives using the “VariantFiltration” function with the following parameters: “QD < 25.0 || FS > 5.0 || MQ < 55.0 || MQRankSum < −0.5 || ReadPosRankSum < −2.0 || SOR > 2.5”. Hard filtering parameters were determined following the “Hard-filtering germline short variants” protocol (https://gatk.broadinstitute.org/hc/en-us/articles/360035890471-Hard-filtering-germline-short-variants). To reduce linkage between SNVs, we used VCFtools v0.1.14 to space SNV markers by a minimum of 3.5 Kb using the following option “–thin 3500”. Phylogenetic analysis of an alignment of 7,386 SNVs was conducted in MEGAX v10.0.5 (Kumar et al., 2018). We used model test in MEGAX to predict the best fit nucleotide substitution model, which was determined as the model with the lowest Bayesian Information Criterion (BIC) value. The general time reversible (GTR) gamma substitution model, with 100 bootstrap replicates, was used to construct the maximum likelihood phylogenetic tree.

### Read Mapping, Variant Detection and Variant Annotation

Quality and adapter trimmed read sets from *A. fumigatus* IP_23, IP_24, IFM47072 and IFM59985 were mapped against the *A. fumigatus* Af293 reference genome (Genbank Assembly Accession number: GCA_000002655.1) (Nierman et al., 2005) using BWA-MEM v0.7.15 (Li and Durbin, 2009). Joint variant calling (SNVs and indels) was conducted using freebayes v1.3.1 with the default settings with the exception of setting ploidy to haploid (–ploidy = 1) (Garrison and Marth, 2012). While the vast majority of indels consisted of 1 or 2 bps (68%), indels as large as 49 bp were identified.

We implemented several filtering steps to prioritize the identification of variants putatively associated with the white-spore phenotype (depicted in **Figure 2**). We identified 51,551 SNVs and 15,183 indels that differed in at least one sample relative to the Af293 reference genome. Next, we narrowed our candidate list by identifying variants that were (i) identical in IP_23 and IP_24, (ii) different from the Af293 genotype, and (iii) different from the IFM47072 and IFM59985 genotypes (4,279 SNVs and 1,785 indels) (**Data Sheet S1**). Finally, we further filtered our candidate variant list by prioritizing variants annotated as “high impact” using SnpEff v4.3t with the *A. fumigatus* Af293 reference genome annotation (Cingolani et al., 2012) (19 SNVs and 45 indels). *Saccharomyces cerevisae* and *Schizosaccharomyces pombe* orthologs of the *A. fumigatus* candidate genes were identified using FungiDB (Stajich et al., 2012; Basenko et al., 2018).

### Analysis of DHN-Melanin Gene Cluster

Because DHN melanin is the molecule responsible for the greenish pigment in wild-type *A. fumigatus* spores, we focused on variants in the DHN melanin encoding gene cluster (Heinekamp et al., 2012). This gene cluster contains 6 genes (*Afu2g17530*, *Afu2g17540*, *Afu2g17550*, *Afu2g17560*, *Afu2g17580* and *Afu2g17600*). We used the “intersect” function in bedtools v2.29.2 (Quinlan and Hall, 2010) to identify SNVs and indels for which genotypes were identical in the white-spore isolates, different from Af293, and different from the green-spore isolates. We focused on *pksP* (*Afu2g17600*) because it contained 3 candidate SNVs (all synonymous variants in the *pksP* gene on chromosome 2: 4687866, 4688908 and 4691782) and 2 candidate indels (one intron variant chromosome 2: 4692683 and one frameshift variant chromosome 2: 4692995) (**Data Sheet 1**).

The *pksP* gene was extracted from the IP_23, IP_24, IFM47072, IFM59985 and Af293 genomes using the samtools “faidx” function (Li et al., 2009). *pksP* sequences were aligned with MAFFT using the default settings (Katoh and Standley, 2013). Alignments were visualized in BioEdit (Hall et al., 2011), variants were visually inspected, and the coding region was extracted and translated. PksP protein domains were predicted in white-spore and green-spore isolates using the PFAM webserver (http://pfam.xfam.org/search/sequence) (El-Gebali et al., 2019).

### Protein Modeling of White-Spore PksP

The Alphafold2 model (Jumper et al., 2021) for *A. fumigatus* PksP was downloaded from EMBL-EBI (https://alphafold.ebi.ac.uk/entry/Q4WZA8) along with the corresponding predicted aligned error (PAE) file. The model was pruned of low confidence residues (pLDDT< 50). The PAE of the deleted domains were then evaluated for inter-residue confidence. Within the truncated region, each of the three terminal domains (two phosphopantetheine attachment site (PP-binding) and one thioesterase) are well supported in their internal residue placement (pLDDT > 70) and structural arrangement (PAE < 5 Å).

Crystal structure of the thioesterase domain of *Aspergillus parasiticus* PksA was downloaded from PDB (3ILS) (Korman et al., 2010). Structural superposition between the crystal structure and the AlphaFold2 predicted thioesterase domain of *A. fumigatus* PksP was performed using Chimera Matchmaker (Meng et al., 2006). Hydrogen bond identification was done in UCSC Chimera (version 1.15, FindHBond), using the rotamer orientation predicted by AlphaFold2. Hydrogen bonds were predicted only between residues having both high absolute confidence (pLDDT > 70) and precise relative placement (PAE < 5 Å).

### Measurement of Growth Rate at 37°C and 48°C

We compared growth rate on minimal media (MM) at 37°C and 48°C, to examine whether our natural albino variants displayed thermotolerance phenotypes, as previously described for albino strains isolated from the Brazilian rainforest (Couger et al., 2018). *Aspergillus* MM was prepared as previously described (Cove, 1966). For each isolate, ~10^5^ spores were inoculated onto the center of MM agar plates and incubated at 37°C for 2 days and 48°C for 3 days in darkness. Experiments were conducted in triplicate and at the conclusion of the experiment colony diameter was measured with digital calipers. Because colony shape is often slightly irregular, we took two random measurements of diameter per plate and averaged these values. Additionally, to assess whether the putative non-functionality of IP_23 and IP_24 PksP contributes to growth patterns at optimal or thermal stress conditions, we performed the same experiment described above, with *A. fumigatus* Af293 and the Af293 *ΔpksP* null mutant (which produces white conidia) generated as previously described (Al Abdallah et al., 2017).

## Results

### Genome Assembly of White-Spore and Green-Spore Isolates

Genome assemblies for IP_23 (386.75), IP_24 (110.46), IFM47072 and IFM59985 were generated with SPAdes (Bankevich et al., 2012). Genome size, as indicated by the cumulative length of the genome assemblies, was highly similar across the four isolates and ranged from 28.36 Mb – 28.80 Mb (Table S1). We used Augustus to predict gene models in each assembly, and similarly, found highly comparable gene numbers (8,855 – 8,908) (Table S1). We used BUSCO to assess genome completeness for each assembly and found that >97% of genes were completely recovered using the eurotiomycetes_odb10 dataset (Simao et al., 2015). These results reflect high quality genome assemblies.

### Phylogenetic Relationship of White Spore Isolates

**Figure 1:**
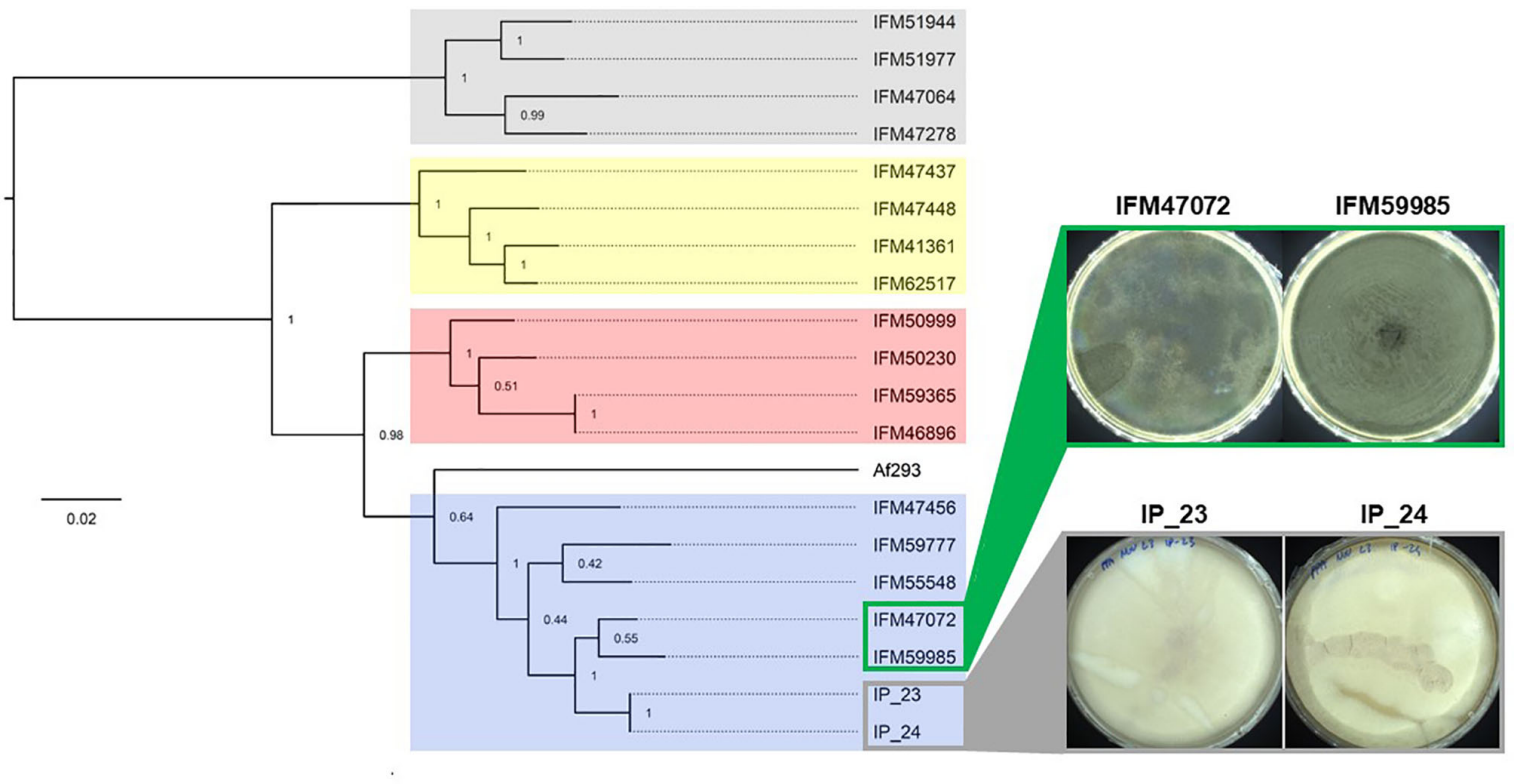
Phylogenetic relationship of white-spore isolates IP_23 and IP_24. Maximum likelihood phylogenetic tree of 7,386 SNVs. The tree is rooted at the midpoint and bootstrap values are provided at each node. Colored clades represent population structure assignments as previously described (Zhao et al., 2021). IP_23 and IP_24, which produce white spores, and IFM4702 and IFM59985, which produce green spores, are members of population 1. Isolates were grown on potato dextrose agar for 7 days at 37°C for imaging.

We previously performed population genomic analysis of 76 clinical *A. fumigatus* isolates from Japan and identified 4 major populations (Zhao et al., 2021). To examine the relationship of IP_23 and IP_24 to these isolates, we conducted phylogenetic analysis with at least 4 individuals from each population. Our analysis again confirmed the presence of 4 populations (**Figure 1**). IP_23 and IP_24 clustered within population 1 and were closely related to IFM47072 and IFM59985 (**Figure 1**). Thus, we focused our comparative genomic analysis on the white-spore isolates IP_23 and IP_24, and the closely related green-spore isolates IFM47072 and IFM59985 (**Figure 1**). We identified 10,950 SNV sites in which IFM47072 and IFM59985 differed in genotype and 2,129 SNV sites in which IP_23 and IP_24 differed in genotype, suggesting IFM47072 and IFM59985 and IP_23 and IP_24 are closely related but not clones.

### Identification of Candidate Variants Associated With White-Spore Phenotype

**Figure 2:**
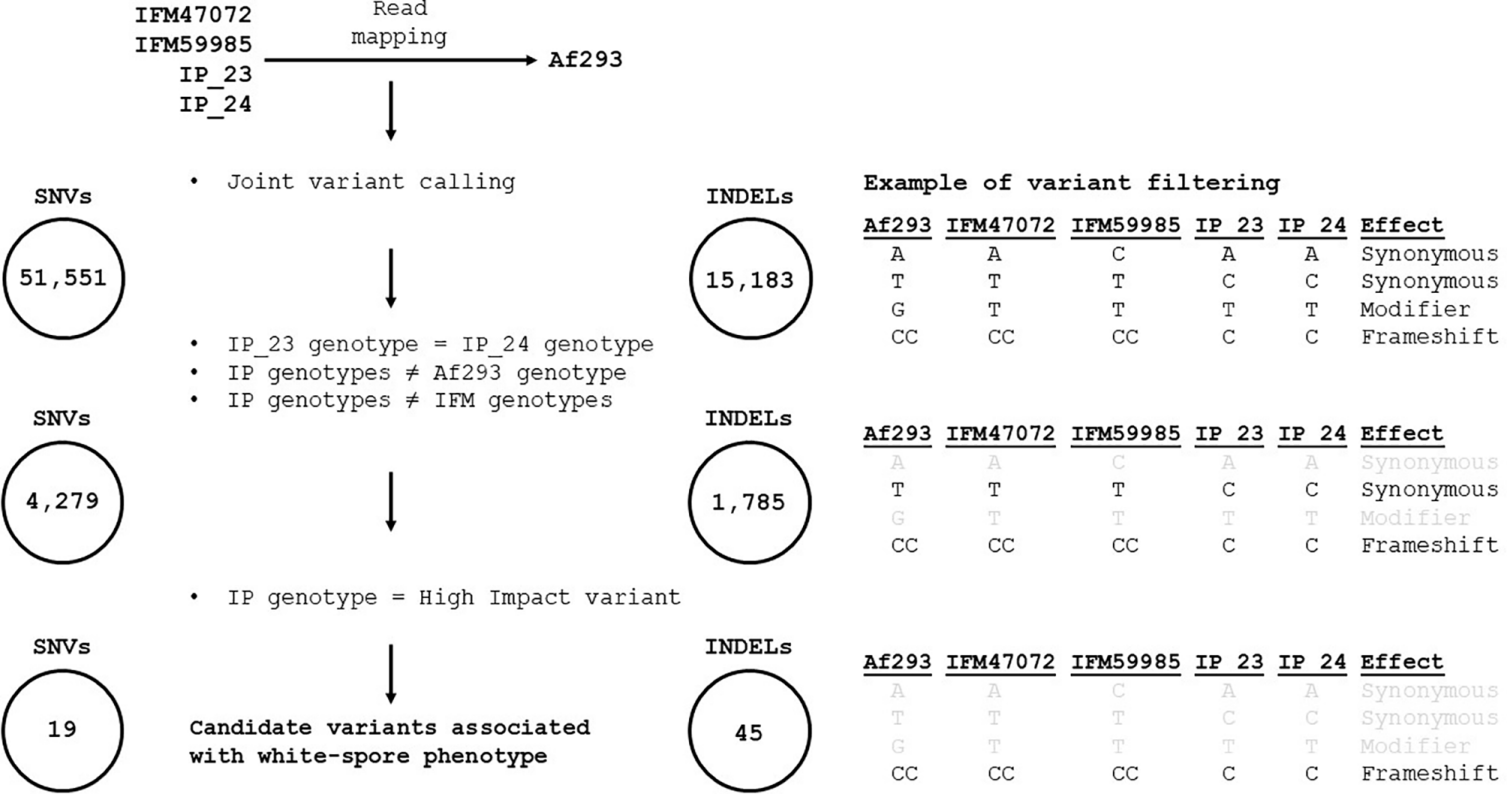
Methodological pipeline to identify candidate variants associated with white-spore phenotype. Reads from IP_23, IP_24, IFM4702 and IFM59985 were independently mapped against the Af293 reference genome and joint genotyping was performed with FreeBayes, resulting in 51,551 SNVs and 15,183 indels. Variants were retained if (i) the variant was identical in IP_23 and IP_24, (ii) the IP_23 and IP_24 variant was different from the Af293 genotype, and (iii) the IP_23 and IP_24 variant was different from the IFM4702 and IFM59985 genotypes (resulting in 4,279 SNVs and 1,785 indels). Lastly, variants were retained if they were annotated as “high impact” by SnpEff. This process resulted in 19 and 45 candidate SNVs and indels, respectively. The right panel provides an example of our filtering scheme. Gray text signifies variants that did not pass the filtering step.

**Figure 3:**
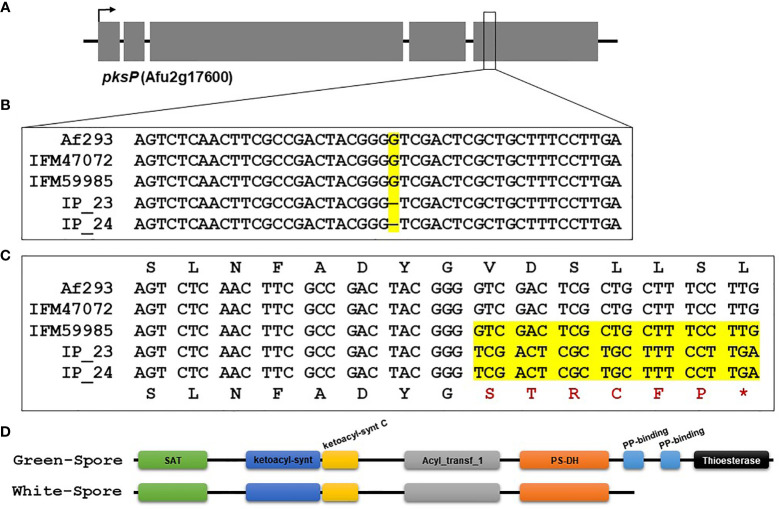
Single nucleotide deletion in pksP results in a truncated protein in IP_23 and IP_24. **(A)** Schematic of the *pksP* gene. The arrow represents the direction of transcription, and grey boxes represent exons. **(B)** Alignment of the region containing the single nucleotide deletion in IP_23 and IP_24 isolates. The yellow region highlights the mutation. **(C)** Translated region containing the single nucleotide deletion. The yellow region shows the single nucleotide deletion, resulting frameshift and premature stop codon (*). **(D)** Protein schematic of PksP in the green-spore and white-spore isolates. Colored ellipses represent PFAM domains. The white-spore PksP is truncated and missing 1 and a half PP-binding domains and the thioesterase domain.

We used FreeBayes to perform joint genotyping of IP_23, IP_24, IFM47072 and IFM59985 relative to the Af293 genome. We identified 51,551 SNVs and 15,183 indels that differed in at least one sample relative to the Af293 reference genome. Next, we narrowed our candidate list by identifying variants that were (i) identical in IP_23 and IP_24, (ii) different from the Af293 genotype, and (iii) different from the IFM47072 and IFM59985 genotypes. This approach yielded 4,279 SNVs and 1,785 indels (**Data Sheet S1**). Next, we predicted the putative functional effects of these candidate variants using SnpEff (Cingolani et al., 2012). We hypothesized that the white-spore phenotype was the result of a loss-of-function mutation and further prioritized candidate variants that were annotated as “High Impact” by SnpEff (i.e., loss of stop codon, gain of stop codon, loss of start codon, splice donor variant, and splice acceptor variant). We identified 19 SNVs and 45 indels annotated as putative high impact mutations (**Figure 2**, **Data Sheet S2**). One of the 64 candidate high impact variants was present in the biosynthetic gene in the DHN-melanin gene cluster (*pksP*, Afu2g17600), and was annotated as a frameshift variant (**Data Sheet S2**). This variant, which is only present in IP_23 and IP_24, is a single nucleotide deletion in the fifth exon of *pksP* and results in a premature stop codon yielding a predicted protein length of 1,686 amino acids in the white-spore isolates compared to 2,148 amino acids in the green-spore isolates (**Figure 3**). To confirm this variant, which was identified through a mapping-based approach, we extracted the *pksP* gene from each genome assembly and aligned the sequences. Again, we observed the single nucleotide deletion at position 5,035.

To gain insight into the functional consequences of the PksP protein truncation, we predicted PFAM protein domains in the green-spore and white-spore PksP proteins. The green-spore PksP contains 8 protein domains (**Figure 3D**). However, the last two-and-a-half PksP protein domains are absent in the white-spore protein. These domains include two consecutive PP-binding domains (PF00550) and a thioesterase domain (PF00975) (**Figure 3D**).

**Figure 4:**
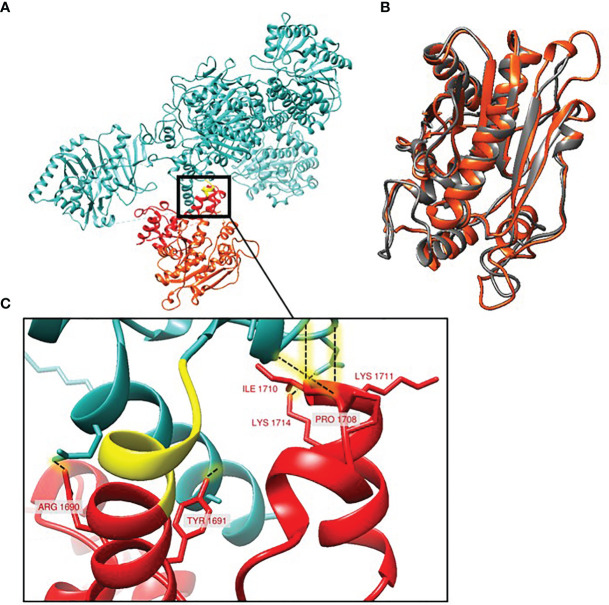
Structural model of *A. fumigatus* PksP protein. **(A)** The conserved region between the green-spore and white-spore isolates (blue) and the region affected by the frameshift variant observed in the white-spore isolates (yellow and red). The yellow region represents the 6 amino acids altered by the frameshift mutation. The red region represents the portion of PksP deleted in the white-spore isolates; the terminal red-orange region corresponds to the predicted thioesterase domain. **(B)** Structural superposition (0.987Å RMSD) between the crystal structure of the thioesterase domain of *Aspergillus parasiticus* PksA (gray) and the predicted *A. fumigatus* PksP thioesterase domain (red-orange). **(C)** Hydrogen bonding (dashed lines) within the first PP domain of PksP that aren’t in the truncated protein.

Structural modeling of the PksP protein using AlphaFold2 (Cramer, 2021) confirms that the truncation removes several highly structured, C-terminal motifs (**Figure 4A**). The largest of the structural motifs predicted by AlphaFold2 superimposes over the crystal structure of the thioesterase domain of *Aspergillus parasiticus* PksA (Korman et al., 2010) to sub-angstrom RMSD (RMSD=0.987Å; **Figure 4B**). The truncation also removes six residues (Lysine 1711, Isoleucine 1710, Threonine 1709, Proline 1708, Tyrosine 1691, Arginine 1690) predicted to participate in hydrogen bonding with residues within the remaining portion of the protein (**Figure 4C**). Thus, the AlphaFold2 model not only corroborates the thioesterase predictions, it also suggests that the truncation may affect conformational stability within the remaining protein through the removal of hydrogen bond partners.

### White-Spore Isolates Are Thermosensitive

**Figure 5:**
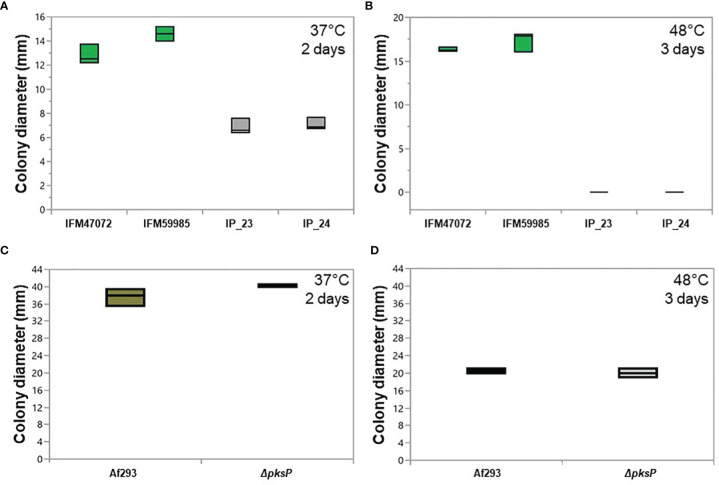
Growth patterns of green-spore and white-spore isolates. Colony diameter of green-spore (green boxplot) and white spore (gray boxplot) isolates after growth at 37°C for 2 days **(A)** and 48°C for 3 days **(B)**. ~10^5^ spores were inoculated in the center of minimal media plates, and colony diameter was measured at the end of the growth period. Each experiment was performed in triplicate. Green-spore isolates grew significantly faster than white-spore isolates at 37°C. White-spore isolates did not grow at 48°C. All statistical tests between green-spore and white-spore isolates (IP_23 vs. IFM47072, IP_23 vs. IFM59985 IP_24 vs. IFM47072 and IP_24 vs. IFM59985) were significant (all p-values ≤ 0.0001). Statistical tests between IP_23 vs. IP_24 and IFM47072 vs. IFM59985 were not significant (p-values > 0.09). Colony diameter of Af293 (green boxplot) and *ΔpksP* (null mutant) (white boxplot) at 37°C for 2 days **(C)** and 48°C for 3 days **(D)**. *ΔpksP* growth rate was not significantly different than Af293 at 37°C (p-value = 0.15) or 48°C (p-value = 0.50).

Couger et al. (2018) isolated an albino strain of *A. fumigatus* from Brazilian rainforest soil that was thermotolerant (Couger et al., 2018). To assess whether IP_23 and IP_24 were also thermotolerant, we compared growth rate on minimal media (MM) at 37°C and 48°C. A one-way ANOVA was performed to compare the effect of temperature on growth rate across green-spore and white-spore isolates, which revealed a significant difference at both temperatures (37°C: F-ratio = 86.8, d.f. = 3, p-value = 6.7e-6 and 48°C: F-ratio = 899.9, d.f. = 3, p-value = 1.9e-10). A *post-hoc* Tukey’s HSD test revealed significant growth rate differences between all green-spore and white-spore comparisons (all p-values ≤ 0.0001) but not between green-spore isolates or white-spore isolates (all p-values ≥ 0.09). The white-spore isolates grew significantly slower at 37°C and 48°C (**Figures 5A, B**). White-spore isolates did not grow at 48°C.

To assess whether PksP functionality directly contributes to the thermosensitivity phenotype in IP_23 and IP_24, we assessed growth rate patterns of *A. fumigatus* Af293 and Af293 *ΔpksP* (Al Abdallah et al., 2017), at 37°C and 48°C (**Figures 5C, D**). We did not observe significant growth rate differences between Af293 and *ΔpksP* for either temperature (**Figures 5C, D**), suggesting that *pksP* is not directly involved in the thermosensitivity phenotype observed in IP_23 and IP_24.

To provide additional insight into variants and/or genes that may contribute to the thermosensitivity phenotype, we identified *S. cerevisiae* orthologs for the 57 candidate genes containing the 64 high impact mutations described earlier (**Figure 2**). We observed a 1 bp insertion resulting in a frameshift in *Afu4g04740* in IP_23 and IP_24 (Chr4: 1,336,071) (**Datasheet 2**). The *S. cerevisiae* ortholog of *Afu4g04740* is *SRM1*, and mutants of *SRM1* (also known as *prp20*) are temperature sensitive (Vijayraghavan et al., 1989; Fleischmann et al., 1991; Clement et al., 2006). In addition, null mutants of *pim1*, the *Schizosaccharomyces pombe* ortholog of *Afu4g04740*, are also thermosensitive (Matsuo et al., 2011).

## Discussion

Here, we investigated the genetic underpinnings of a white-spore phenotype in two natural isolates of *A. fumigatus* by comparing their genomes to two closely related isolates with the wild-type green-spore phenotype (**Figure 1**). We identified 64 candidate variants that were identical in the white-spore genomes and different in the Af293 reference genome and the closely related genomes of the green-spore isolates (**Figure 2**, **Data Sheet S2**). One of these candidate variants was a single nucleotide deletion in the *pksP* gene which results in a frameshift mutation and a truncated PksP protein (**Figures 3**, **4**). We also observed that white-spore isolates were thermosensitive, and provide insight into a variant in a candidate gene that may influence this phenotype (**Figure 5**).

Contrary to a previous study, which observed increased thermotolerance in a natural white-spore isolate of *A. fumigatus* (Couger et al., 2018), IP_23 and IP_24 were thermosensitive compared to green-spore isolates (**Figures 5A, B**). First, we explored whether the putatively non-functional copy of PksP in IP_23 and IP_24 was driving this phenotype. We performed growth rate analysis of *A. fumigatus* Af293 and a null *pksP* mutant in the Af293 background. We did not observe a difference in growth rate between Af293 and *ΔpksP* at 37°C or 48°C (**Figures 5C, D**), which suggests PksP is not involved in the thermosensitivity phenotype. Next, we examined the high impact candidate variants that are shared in IP_23 and IP_24 and different from Af293, IFM4702 and IFM59985. We identified one gene, *Afu4g04740*, that contained a frameshift variant for which deletion mutants in the *S. cerevisave* and *S. pombe* orthologs displayed temperature sensitivity phenotypes (Vijayraghavan et al., 1989; Fleischmann et al., 1991; Clement et al., 2006; Matsuo et al., 2011). *SRM1*, the *S. cerevisae* ortholog of *Afu4g04740*, enables guanyl-nucleotide exchange factor activity. *SRM1* null mutants in *S. cerevisae* and *S. pombe*, display reduced growth at high temperatures (Clement et al., 2006; Matsuo et al., 2011). While it is clear that *SRM1* interacts at specific chromatin regions allowing nuclear pore complexes to modify chromatin organization (Dilworth et al., 2005), the mechanism between *SRM1* and temperature sensitivity is less apparent. In light of these results, *Afu4g04740* represents an interesting future candidate gene to target in *A. fumigatus* to assess its role in thermosensitivity.

The frameshift mutation in *pksP* results in the loss the one and a half phosphopantetheine attachment site (PP-binding) domains and a thioesterase domain (**Figure 3**). The PP-binding domains are involved in transporting the secondary metabolite substrate and chain intermediates to the catalytic centers during the biosynthesis of polyketides (Pihet et al., 2009). Interestingly, a portion of the *pksP* PP-binding domain in *Aspergillus luchuensis* was also deleted in industrial and artificially mutated albino strains compared to strains with wild-type spore color, which suggests a critical functional role for this domain in PksP function (Yamamoto et al., 2021). Additionally, previous studies have established the essentiality of the thioesterase domain in PksP for naphthopyrone synthesis (Vagstad et al., 2012). Specifically, the thioesterase domain catalyzes bond formation between C2 and C7 during the biosynthesis of 1,3,6,8-tetrahydroxynaphthalene (THN) (Vagstad et al., 2012). In agreement with our findings, a previous study identified a nonsense mutation and independent frameshift mutations in white-spore isolates of *A. fumigatus* that led to protein truncations and losses of the (i) PP-binding domains and the thioesterase domain, and (ii) the thioesterase domain (Pihet et al., 2009).

Recent genomic analysis of an albino *A. fumigatus* strain isolated from the Brazillian rainforest was conducted (Couger et al., 2018). Analysis of the DHN-melanin gene cluster revealed interruption of the cluster by a ~28 Kb region of fungal and unknown origin. PCR amplification of conserved regions in wild-type *A. fumigatus* isolates suggested additional structural variation in the *pksP* region. All genes in the *pskP* gene cluster were either lowly expressed or not expressed (Couger et al., 2018). Thus, convergent mutations in *pksP* can lead to the loss of conidia pigmentation.

Our results, and the work of others (Sarfati et al., 2002; Balajee et al., 2004; Couger et al., 2018), suggest that the loss of spore pigmentation is naturally variable in *A. fumigatus*. Understanding the ecology driving these patterns of variation is an important step in determining why albinism may be maintained in natural populations. For instance, DHN-melanin is involved in protecting the cell from ultra-violet (UV) radiation, and it is conceivable to hypothesize that selective pressure maintaining DHN-melanin production may be relaxed in environments where UV exposure is reduced. An ecological survey of UV-B radiation revealed that levels were significantly lower near the forest floor compared to the outer canopy (Brown et al., 1994). Thus, it would be interesting to investigate whether rates of albinism in *A. fumigatus* are elevated in environments where UV radiation is relatively low. Relaxed selection for loss of protective pigmentation in low UV environments has occurred in a variety of organisms including humans (Jablonski, 2004), coconut crabs (Caro, 2021), and many cave animals (Protas et al., 2006; Protas et al., 2011; Bilandzija et al., 2012). Interestingly, DHN-melanin deficient mutants of two clinical isolates of *A. fumigatus* were more sensitive to UV-C radiation (Blachowicz et al., 2020). However, the DHN-melanin deficient mutant of a strain collected from the International Space Station, where UV-C radiation is elevated, was not more sensitive to UV-C. This observation suggests mechanisms independent from DHN-melanin can contribute to UV-C tolerance.

Our study demonstrates the utility of using comparative genomic analysis on closely related but phenotypically distinct isolates. This approach could be further applied to identify genes governing stress resistance or virulence, phenotypes which have been repeatedly demonstrated to be variable among isolates of *A. fumigatus* (Mondon et al., 1996; Ben-Ami et al., 2010; Alshareef and Robson, 2014; Kowalski et al., 2016; Keizer et al., 2021). Our comparative genomics approach (**Figure 2**) could also be applied to chemical mutagenesis screens in which the genomes of phenotypically distinct mutants are directly compared to their parental strains (Bok et al., 2014; Li et al., 2016), and evolve and resequence experiments in which an isolate is continuously cultured in a controlled environment and the genome sequence of the adapted lineage is directly compared to the parental strain (Long et al., 2015; Zhang et al., 2017).

## Data Availability Statement

The datasets presented in this study can be found in online repositories. The names of the repository/repositories and accession number(s) can be found below: https://www.ncbi.nlm.nih.gov/, SRR16944308 https://www.ncbi.nlm.nih.gov/, SRR16944307.

## Author Contributions

JGG oversaw the project, designed the analysis, carried out some of the genomic analysis, and lead the manuscript writing. PD conducted genomic analysis and edited the manuscript. SZ sequenced the genomes, conducted some genomic analysis, conducted growth rate analysis and edited the manuscript. GWX conducted growth rate analysis. DCR conducted protein modeting analysis and edited the manuscript. JRF generated the pksP mutant and edited the manuscript. JPL provided strains and edited the manuscript. All authors contributed to the article and approved the submitted version.

## Funding

This research was supported by grant R21AI137485 from the National Institutes of Health and National Institutes of Allergy and Infectious Diseases (NIAID) to JG which supported JG and SZ.

## Conflict of Interest

The authors declare that the research was conducted in the absence of any commercial or financial relationships that could be construed as a potential conflict of interest.

## Publisher’s Note

All claims expressed in this article are solely those of the authors and do not necessarily represent those of their affiliated organizations, or those of the publisher, the editors and the reviewers. Any product that may be evaluated in this article, or claim that may be made by its manufacturer, is not guaranteed or endorsed by the publisher.
