# Supplementary material for: Comparative Genomics Reveals a Single Nucleotide Deletion in pksP That Results in White-Spore Phenotype in Natural Variants of Aspergillus fumigatus
Source: Front Fungal Biol. 2022 Jun 7;3:897954. doi: 10.3389/ffunb.2022.897954 (PMC10512363; doi:10.3389/ffunb.2022.897954)
Supplement: Supplementary file 3 [file Table_1.docx]

**Table S1. Genome assembly statistics.**

|  | **IFM47072** | **IFM59985** | **IP_23** | **IP_24** |
| --- | --- | --- | --- | --- |
| **Scaffolds** | 602 | 295 | 682 | 457 |
| **Cumulative length** | 28,803,730 | 28,366,669 | 28,659,403 | 28,648,064 |
| **Max scaffold length** | 2,477,843 | 2,325,150 | 1,395,233 | 1,801,965 |
| **N50** | 505,485 | 832,276 | 561,740 | 739,817 |
| **Gene models** | 8,887 | 8,855 | 8,908 | 8,888 |
| **BUSCO complete genes (Eurotiomycetes_odb10 db)** | 97.83% | 97.24% | 97.29% | 97.26% |
